# Antihyperlipidemic and hepatoprotective properties of alkali- and enzyme-extractable polysaccharides by *Dictyophora indusiata*

**DOI:** 10.1038/s41598-019-50717-9

**Published:** 2019-10-03

**Authors:** Wenshuai Wang, Honghong Liu, Yiwen Zhang, Yanbo Feng, Fangfang Yuan, Xinling Song, Zheng Gao, Jianjun Zhang, Zhen Song, Le Jia

**Affiliations:** 1 Institute Environment and Sustainable Development in Agriculture, Chinese Academy of Agricultural Sciences, Beijing, 100081 P.R. China; 2 College of Life Science, Shandong Agricultural University, Taian, 271018 P.R. China; 3 Xi’an Aeronautical Polytechnic Institute, Xi’an, 710089 P.R. China

**Keywords:** Dietary carbohydrates, Fungal biology

## Abstract

Hyperlipidemia, a very common disease throughout the world, usually gives rise to severe liver damages. The current experiment was to investigate the antihyperlipidemic and hepatoprotective properties of alkali- and enzyme-extractable *Dictyophora indusiata* polysaccharides (Al-DPS and En-DPS) on the hyperlipidemic mice. The results of animal experiment *in vivo* showed that treatment with Al-DPS or En-DPS could improve the excessive level of lipid profiles in serum and liver, as well as strengthen antioxidant status. In addition, the histopathological observations of liver testified that polysaccharides were capable of attenuating hepatic cell injury. The primary structural features of Al-DPS and En-DPS were demonstrated by HPGPC, HPLC, FT-IR and NMR. Glucose tolerance test manifested that polysaccharides were able to restrain the rise of blood sugar. The results indicated that Al-DPS and En-DPS may be considered as novel compounds to treat hyperlipidemia and also act as hepatoprotective agents.

## Introduction

Hyperlipidemia is a systemic disease, which is characterized by elevated lipid levels in blood including total cholesterol (TC), total glyceride (TG), and low-density lipoprotein cholesterol (LDL-C) and so on^1^. In addition, it is an important risk factor leading to fatty liver, cardiovascular disease and atherosclerosis^2^, and becomes the first killer of human health. It has been reported that nearly 23.6 million people will die from cardiovascular diseases originating from hyperlipidemia by 2030^3^. Furthermore, the incidence of hyperlipidemia is increasing gradually, and may aggravate with aged tendency of population^4^. Hence, the prevention and management of hyperlipidemia is important. Previous studies have indicated that oxidative stress from reactive oxygen species (ROS) could accelerate the pathogenic progress of hyperlipidemia and its complications^5,6^. Increasing evidences have suggested that the hepatoprotective effects of substance may be related to their known antioxidant and pre-oxidant properties^7^. To date, many hypolipidemic agents were commonly used to treat hyperlipidemia including atorvastatin, fibrates, statins, nicotinic acid, probucol and other compound preparations clinically^8^. However, the application of these drugs is limited as a result of their serious side effects, such as diarrhea, nausea, myositis, and abnormal liver functions of statins^9^. Thus, it is eager to exploit natural alternative components possessing antioxidant and hepatoprotection effects aiming to reduce damages linked to hyperlipidemia.

Polysaccharide, an important natural bio-macromolecule, is widely found in nature^10^. In recent years, numerous studies have shown that many natural polysaccharides from mushrooms have been proved to possess potential antihyperlipidemic activities^11–13^. *Dictyophora indusiata*, a kind of famous edible mushroom, is widely distributed and eaten around the world. It is designated as the queen of the mushrooms rich in various nutrients including polysaccharide, protein, mineral, vitamin, amino acids, riboflavin and nicotinic acid^14,15^. For the past few years, polysaccharide extracted from *D*. *indusiata* has attracted great attention because of a number of pharmacological functions. For instance, Deng *et al*. separated a polysaccharide from *D*. *indusiata* and investigated its molecular mechanism involved in the immunostimulatory activity on RAW264.7 cells. Results indicated that TLR4 is the major receptor responsible for the interaction of polysaccharide and macrophages^14^. A regenerated triple helical polysaccharide isolated from *D*. *indusiata* was reported to have effects on inhibiting tumor growth *in vivo* and this antitumor activity maybe associated to immunostimulation^16^. However, few reports focused on the relationships between polysaccharides from *D*. *indusiata* and anti-hyperlipidemia activities. Many evidences demonstrated that high-fat emulsion could resoundingly induce hyperlipidemia in researches^7^.

In the experiment, we adopted aqueous alkali and enzyme solution to separate two kinds of polysaccharides from *D*. *indusiata* fruiting bodies. Meanwhile, the structures of alkali- and enzyme-extractable *Dictyophora indusiata* polysaccharides (Al-DPS and En-DPS) were preliminarily explored, and the animal experiment was performed to investigate the hypolipidemic and hepatoprotective effect of polysaccharides subsequently.

## Results

### Acute toxicity analysis

**Figure 1:**
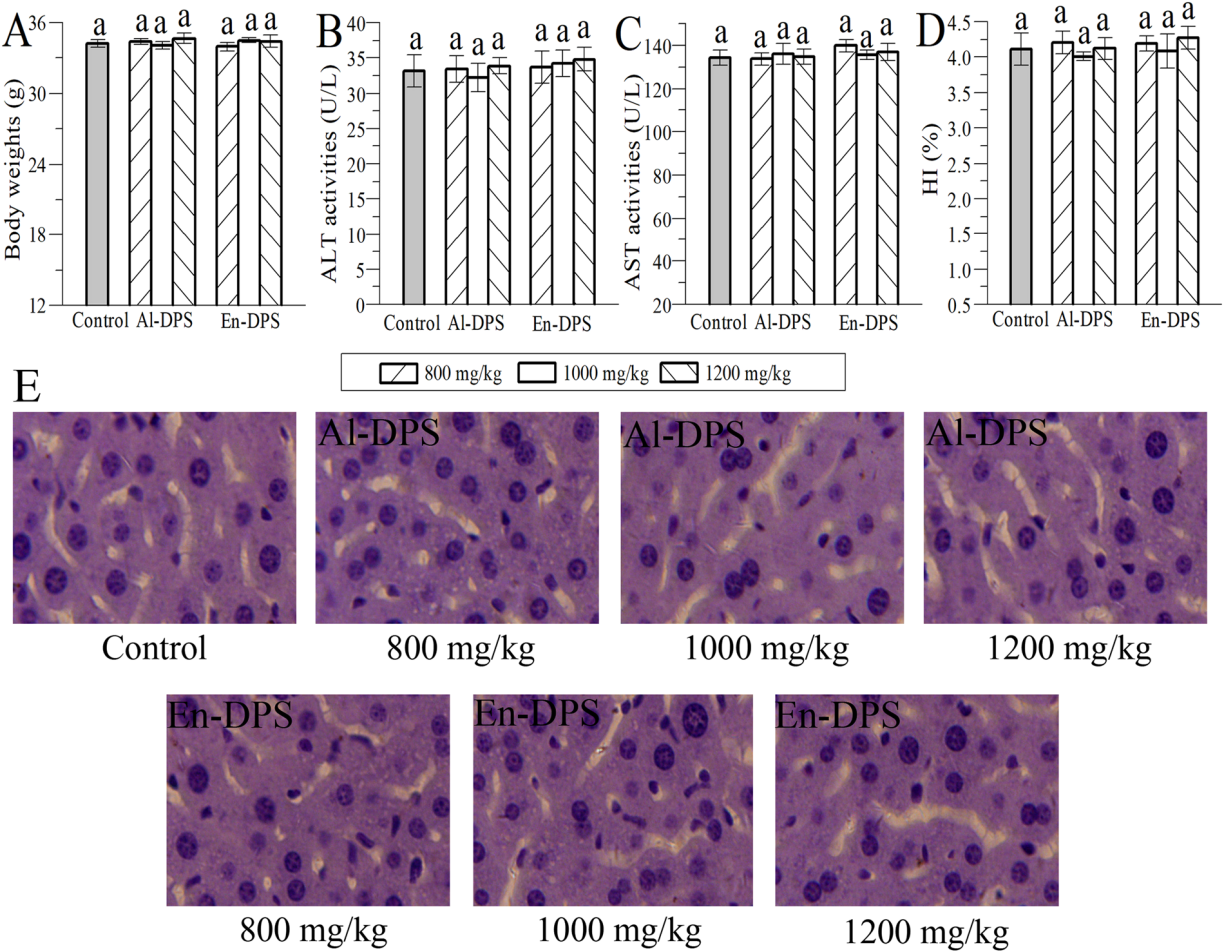
Acute toxicity study results of mice fed Al-DPS and En-DPS. (**A**) Body weights, (**B**) ALT, (**C**) AST, (**D**) HI and (**E**) histopathological observation of liver. Means with the same letter are not significantly different.

The mice treated with Al-DPS or En-DPS did not exhibit any gross behavioral changes, toxic responses or deaths even at a dose of 1200 mg/kg during the feeding period in comparison with control group. Besides, there was no significant difference in the body weights, biochemical analysis (ALT and AST), hepatosomatic index and histopathological observation of liver between dosage group and control group (Fig. 1). In addition, general morphological of liver was normal. The above results manifested that Al-DPS and En-DPS were practically non-toxic substances.

### Effects on body weight and HI

In present work, the yield of Al-DPS and En-DPS were 4.85 ± 0.18% and 4.01 ± 0.22%, respectively. As shown in Table 1, there was no significant difference in initial body weight among all the groups. After 33 days, the body weight in MC group was notably higher than that in NC group due to high-fat emulsion. However, all the polysaccharide groups and PC group showed lower weight compared with MC group, indicating Al-DPS, En-DPS and simvastatin had potential contributions in reducing the gain of weight. As for HI, it was higher in MC group than all the other groups, whereas obvious decline was observed after the supplement of two polysaccharides, especially En-DPS.

**Table 1:** Effects on body weight and HI.

Groups	Body weight (g)		HI (%)
	Initial	Final	
NC	25.12 ± 0.39	34.04 ± 0.27^d^	4.31 ± 0.16^d^
MC	24.97 ± 0.31	41.63 ± 0.36^a^	5.97 ± 0.23^a^
PC	25.27 ± 0.52	35.71 ± 0.25^c^	4.91 ± 0.19^c^
Al-DPS			
400 mg/kg/d	25.33 ± 0.45	36.29 ± 0.48^c^	5.07 ± 0.14^b,c^
200 mg/kg/d	24.94 ± 0.31	37.53 ± 0.39^b^	5.31 ± 0.09^b^
En-DPS			
400 mg/kg/d	25.02 ± 0.40	35.65 ± 0.45^c^	4.93 ± 0.16^c^
200 mg/kg/d	25.15 ± 0.27	37.09 ± 0.53^b^	5.08 ± 0.19^b,c^

The values were reported as the Mean ± S.D. (n = 10 for each group). Means with the same letter are not significantly different.

### Effects on serum lipid levels

**Figure 2:**
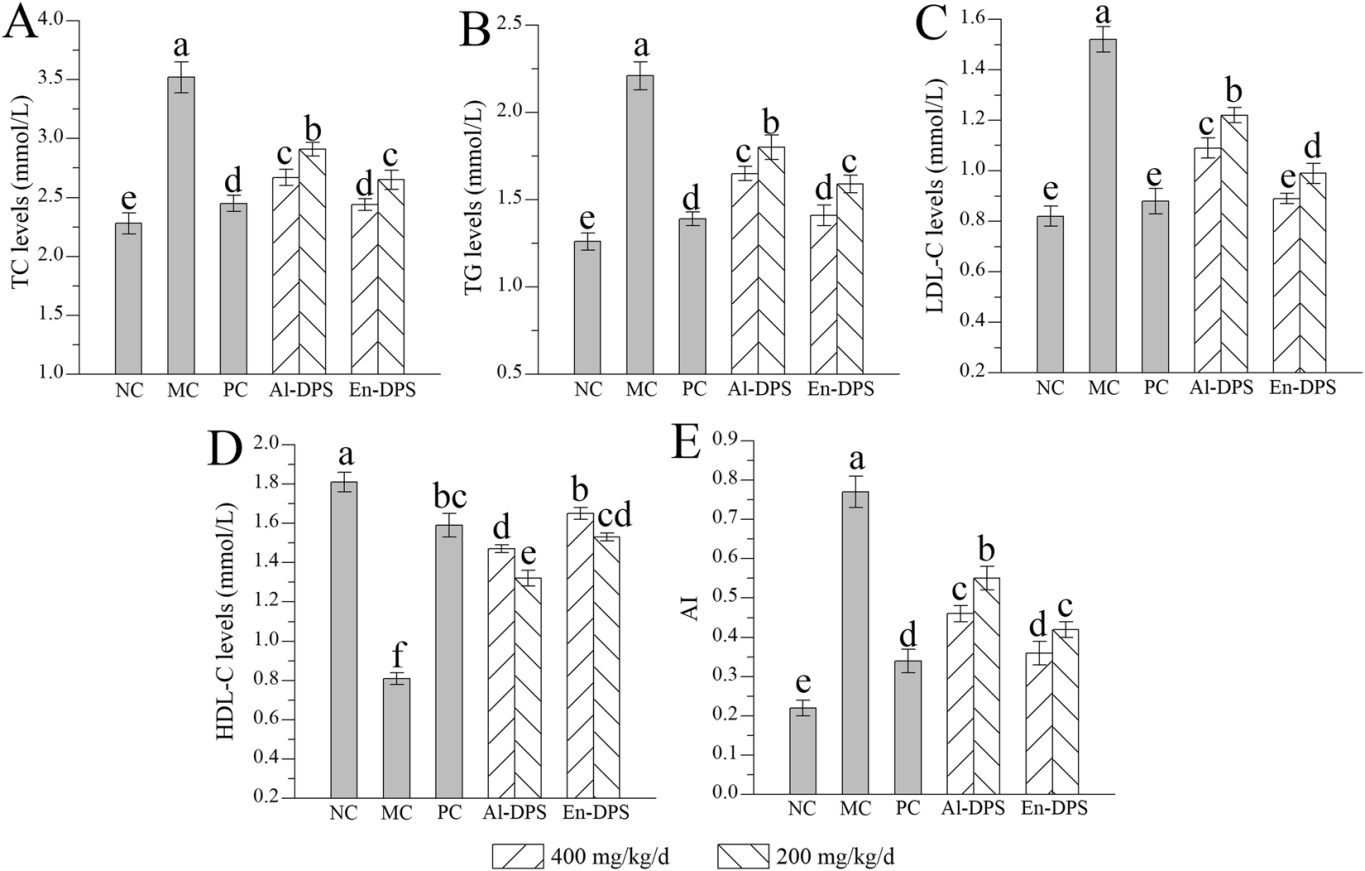
Effects on serum lipid levels. (**A**) TC, (**B**) TG, (**C**) LDL-C, (**D**) HDL-C and (**E**) AI. The values were reported as the Mean ± S.D. (n = 10 for each group). Means with the same letter are not significantly different.

According to Fig. 2, TC, TG, LDL-C and AI levels were higher and the HDL-C level was lower in MC group than those in the NC group, which showed that the hyperlipidemic model of mice was successful. After administration of Al-DPS or En-DPS at a high dose of 400 mg/kg/d, the levels of TC, TG, LDL-C and AI were decreased to 2.67 ± 0.07 mmol/L, 1.65 ± 0.04 mmol/L, 1.09 ± 0.04 mmol/L and 0.46 ± 0.02 as well as 2.44 ± 0.05 mmol/L, 1.41 ± 0.06 mmol/L, 0.89 ± 0.02 mmol/L and 0.36 ± 0.03, which were all lower than that in the MC group. At the same time, the HDL-C in serum was increased by 81.48 ± 0.26% or 103.70 ± 0.14% after treatment with Al-DPS or En-DPS at high dosage compared with the model control group.

### Effects on hepatic lipid levels

**Figure 3:**
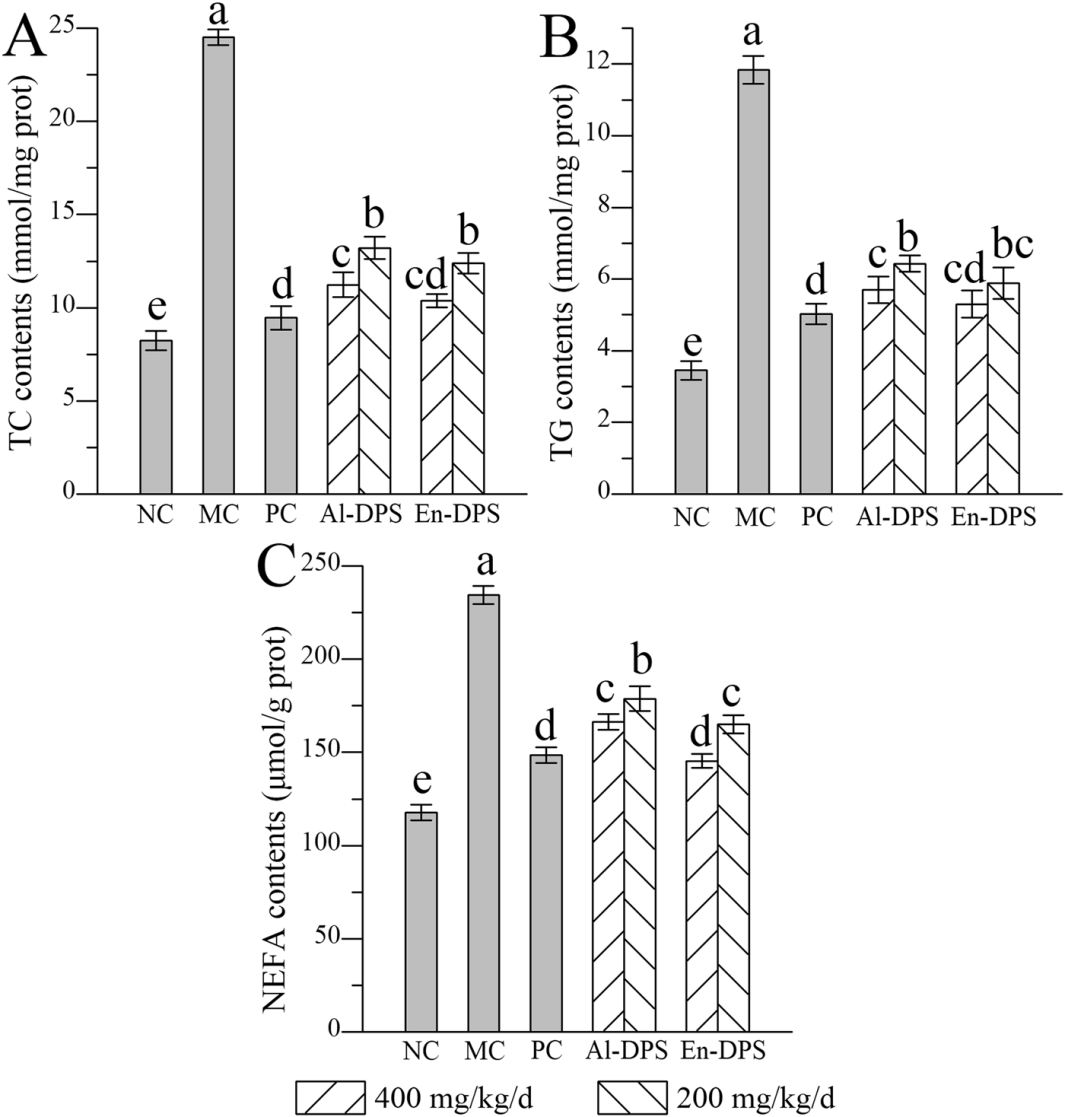
Effects on hepatic lipid levels. (**A**) TC, (**B**) TG and (**C**) NEFA. The values were reported as the Mean ± S.D. (n = 10 for each group). Means with the same letter are not significantly different.

Hepatic lipid levels including TC, TG and NEFA increased visibly in the MC group when compared with that in the NC group, which were the indications of hepatocyte damages (Fig. 3). Significant decreases of hepatic lipid levels in the dosage groups whether 400 or 200 mg/kg/d appeared in comparison with those of the model group, testifying Al-DPS and En-DPS could fight against liver injury.

### Effects on blood glucose level

**Figure 4:**
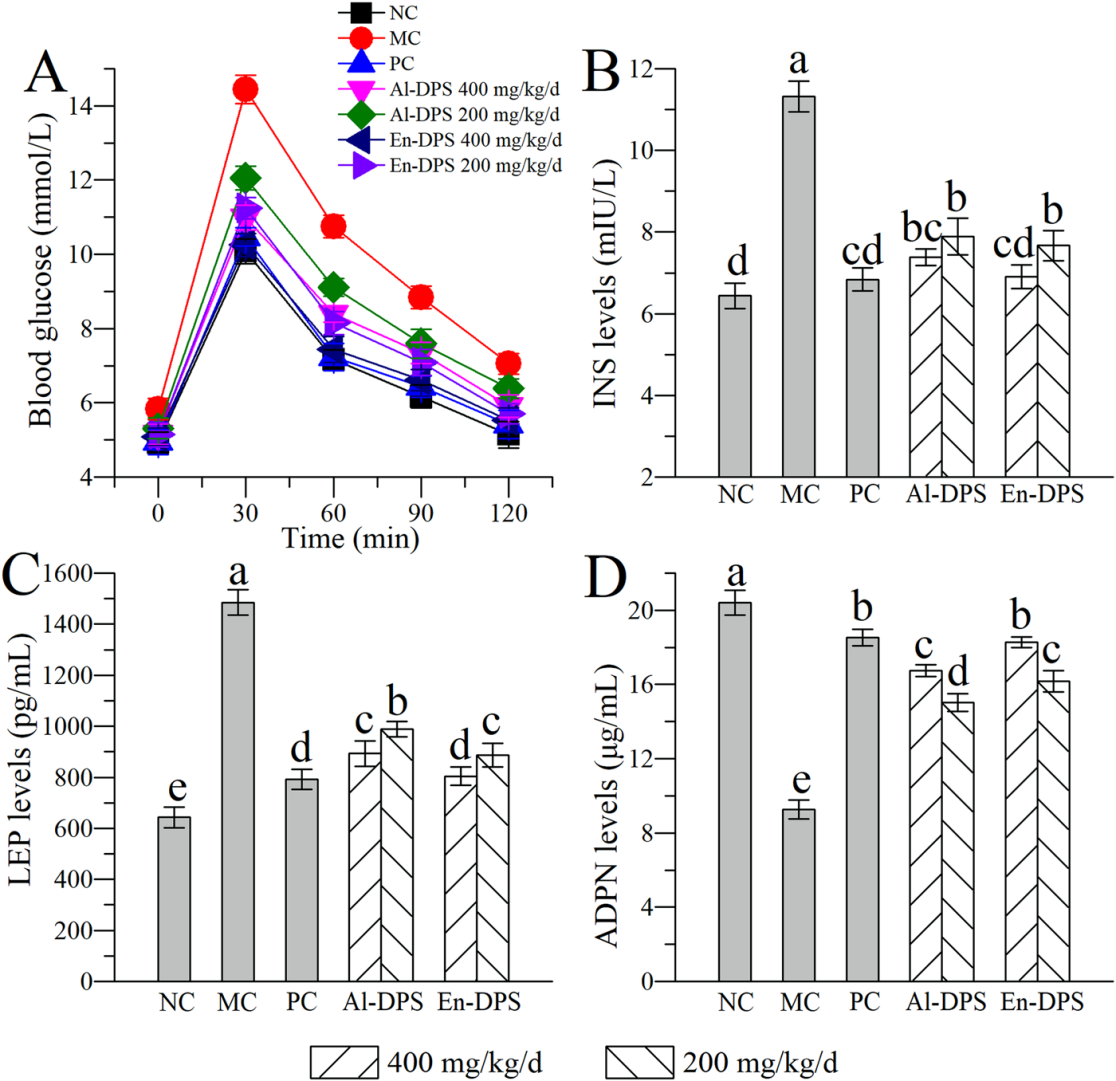
Effects on blood glucose level and the contents of INS, LEP and ADPN. (**A**) Glucose tolerance test, (**B**) INS, (**C**) LEP and (**D**) ADPN. The values were reported as the Mean ± S.D. (n = 10 for each group). Means with the same letter are not significantly different.

As demonstrated in Fig. 4A, the blood sugar rose rapidly and peaked at 30 min. As time went on, it declined gradually. The blood glucose level of MC group was higher than NC group, which made clear that the ability to sense and inhibit the rise of blood sugar was reduced in hyperlipidemic mice. Meanwhile, the blood glucose of polysaccharide groups was lower when compared with MC group, suggesting that Al-DPS and En-DPS were able to inhibit the increase of blood glucose, especially in the high dose group.

### Effects on the contents of INS, LEP and ADPN

In this study, the levels of INS, LEP and ADPN were also examined (Fig. 4B–D). High-fat emulsion resulted in the decrease in ADPN level and the increases in INS and LEP in serum. It was remarkable that the addition of Al-DPS and En-DPS with two dosages alleviated the situation effectively. In addition, the mice treated with En-DPS at 400 mg/kg/d worked best when compared with the mice in MC group.

### Effects on serum enzyme activities and TBIL level

**Figure 5:**
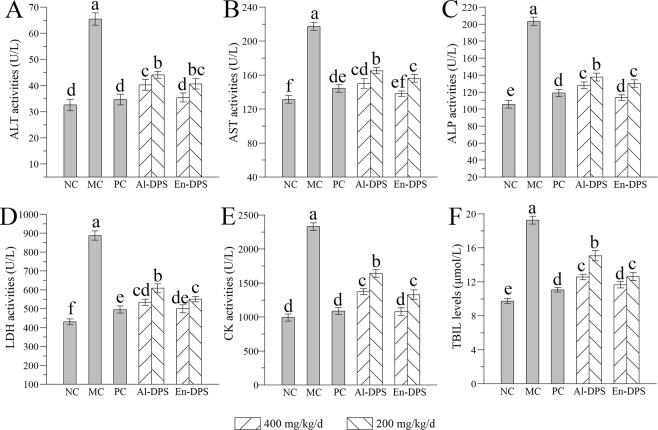
Effects on serum enzyme activities and TBIL level. (**A**) ALT, (**B**) AST, (**C**) ALP, (**D**) LDH, (**E**) CK and (**F**) TBIL. The values were reported as the Mean ± S.D. (n = 10 for each group). Means with the same letter are not significantly different.

In order to analyze the liver protection effect of Al-DPS and En-DPS on hyperlipidemia, we measured the serum enzyme activities (ALT, AST, ALP, LDH and CK) and TBIL level, and the results were displayed in Fig. 5. When compared with NC group, the activities of serum enzymes and the content of TBIL were enhancive observably in hyperlipidemic mice (MC group) and this was a sign of liver damage. Thankfully, significant reductions of above indices were observed. Especially in the high-dose group of En-DPS, the values of ALT, AST, ALP, LDH, CK and TBIL were decreased by 45.87 ± 1.02%, 36.46 ± 1.99%, 44.25 ± 2.11%, 43.70 ± 3.24%, 53.66 ± 3.12% and 39.43 ± 1.04% compared with those of the MC group, implying that En-DPS had prominent protection effects on the hepatic damages induced by hyperlipidemia.

### Effects on hepatic antioxidant activities

**Figure 6:**
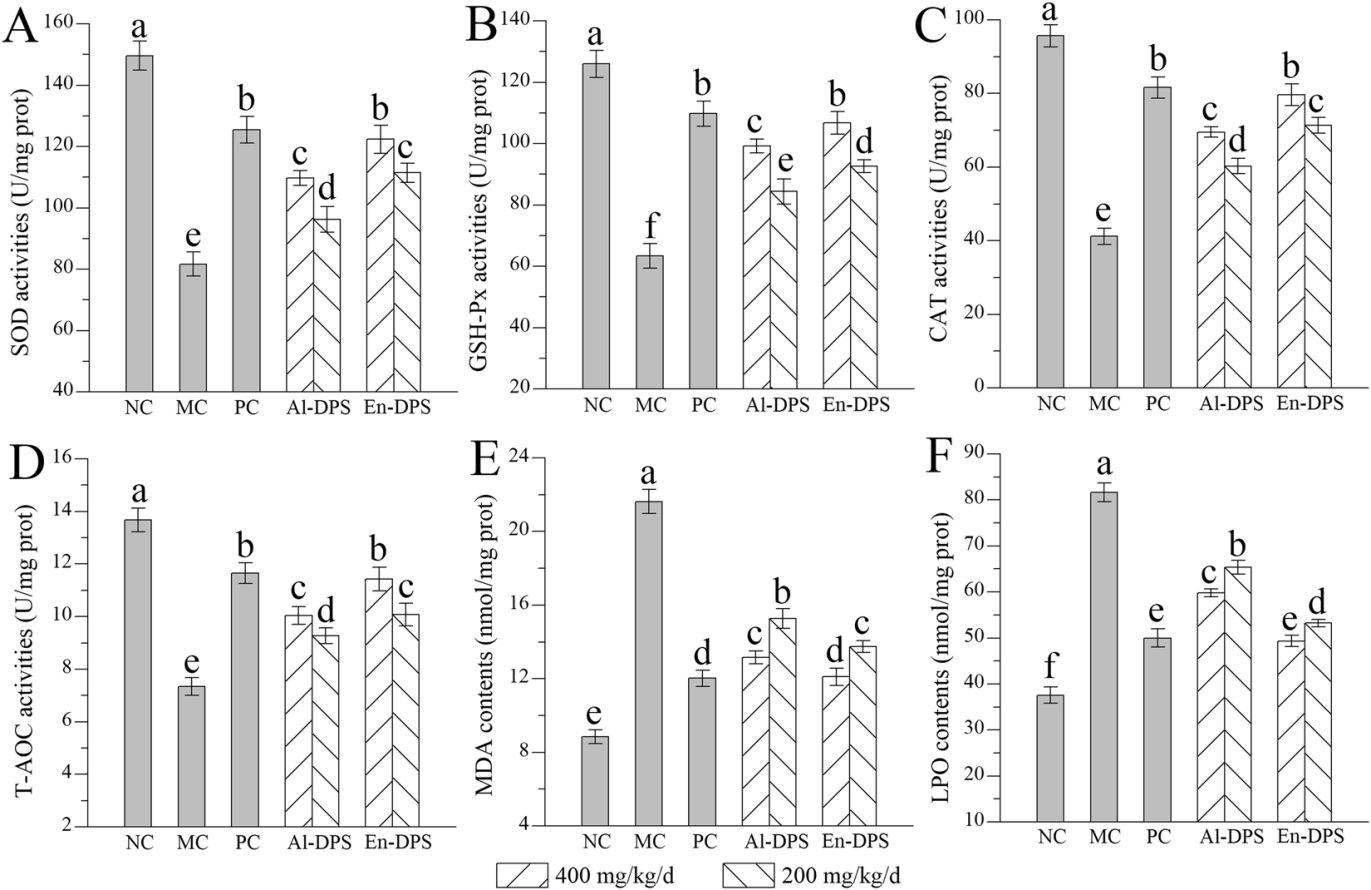
Effects on hepatic antioxidant activities. (**A**) SOD, (**B**) GSH-Px, (**C**) CAT, (**D**) T-AOC, (**E**) MDA and (**F**) LPO The values were reported as the Mean ± S.D. (n = 10 for each group). Means with the same letter are not significantly different.

Antioxidant activities of liver in different groups were showed in Fig. 6. From the figure, decreased antioxidant enzyme activities (SOD, GSH-Px and CAT), reduced non-enzymatic antioxidant capacity (T-AOC), as well as increased lipid product contents (MDA and LPO) were observed in hyperlipidemic mice in comparison with that in the NC group, indicating that serious oxidative stress occurred. Interestingly, these pathological changes could be attenuated by supplementation of Al-DPS or En-DPS.

After En-DPS administration (400 mg/kg/d), the activities of SOD, GSH-Px, CAT and T-AOC (Fig. 6A–D) reached 122.37 ± 4.56 U/mg prot, 106.76 ± 3.67 U/mg prot, 79.63 ± 2.94 U/mg prot and 11.43 ± 0.45 U/mg prot, which were 49.85 ± 2.12%, 68.36 ± 3.23%, 93.23 ± 2.22% and 55.72 ± 3.19% higher than those of MC group, while these activities were increased by 34.33 ± 3.05%, 56.52 ± 4.32%, 68.62 ± 2.36% and 36.78 ± 4.23%, respectively, when compared with the MC group after Al-DPS administration (400 mg/kg/d). The contents of lipid peroxides including MDA and LPO (Fig. 6E,F) were reduced by 44.06 ± 0.31% and 39.55 ± 0.41% in En-DPS-treated mice at 400 mg/kg/d as well as 39.16 ± 0.35% and 26.76 ± 0.22% in Al-DPS-treated mice at 400 mg/kg/d compared with MC group. The results indicated that both Al-DPS and En-DPS successfully suppressed hepatic oxidative damage.

### Liver histopathological observation

**Figure 7:**
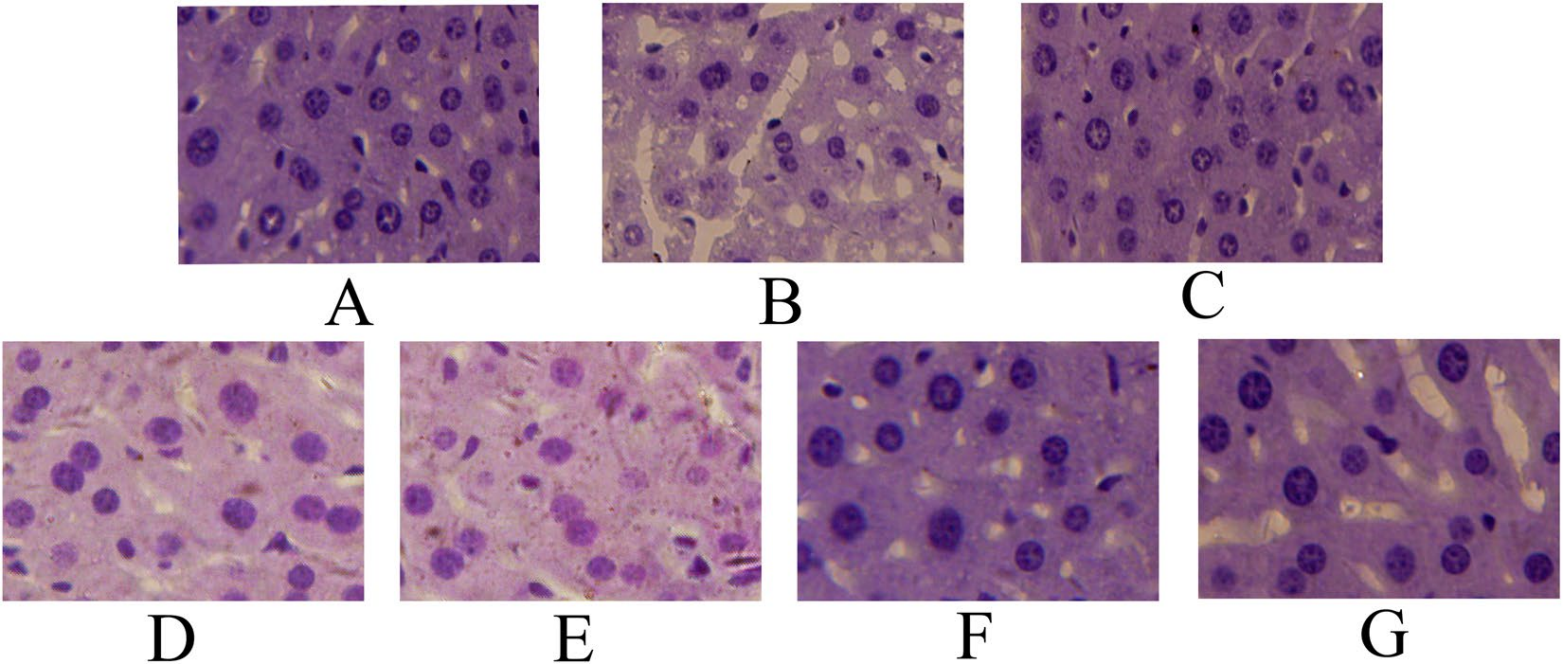
Liver histopathological observation. (**A**) NC groups, (**B**) MC groups, (**C**) PC groups, (**D**) Al-DPS group at 400 mg/kg/d, (**E**) Al-DPS group at 200 mg/kg/d, (**F**) En-DPS group at 400 mg/kg/d and (**G**) En-DPS group at 200 mg/kg/d.

In the current work, the histopathological observations of liver were performed by H&E staining (Fig. 7). The hyperlipidemic mice in MC group showed serious liver damages characterized by loss of cell borders, cell swelling and accumulations of fat differing from typical hepatic cells in NC group with regular hepatocyte morphology, well-defined cell borders and distinct hepatic nucleus. Nevertheless, Al-DPS and En-DPS showed potential effects on preventing liver damages on the basis of Fig. 7D–G. Moreover, the En-DPS recovered morphological structure and degeneration of hepatocytes better than Al-DPS at same dosage.

### Chemical characterizations

The molecular weights of Al-DPS and En-DPS were analyzed by HPGPC and the results were showed in Table 2. It can be seen that the average molecular weights (Mw) of Al-DPS and En-DPS were 5.69 × 10^5^ and 3.82 × 10^5^ Da, respectively. Furthermore, the results of HPLC revealed that Al-DPS was made up of nine kinds of monosaccharides and glucose was the most abundant. En-DPS which contained ten kinds of monosaccharides was mainly composed of galactose. Compared with Al-DPS, a little of galacturonic acid was found in the En-DPS. The detailed percentage compositions of monosaccharides were shown in Table 2.

**Table 2:** The molecular weight and monosaccharide composition of Al-DPS and En-DPS.

Samples	Al-DPS	En-DPS
Mw (×10^5^ Da)	5.69	3.82
Mn (×10^5^ Da)	3.62	2.33
Mz (×10^5^ Da)	7.68	5.26
Mw/Mn	1.57	1.64
Monosaccharide composition (%)		
Mannose	7.58	14.71
Ribose	6.43	1.76
Rhamnose	0.51	0.29
Glucuronic acid	0.43	1.11
Galacturonic acid	—	0.13
Glucosamine	—	—
Glucose	53.47	17.30
Galactosamine	—	—
Galactose	25.57	56.28
Xylose	0.64	0.55
Arabinose	0.19	0.19
Fucose	5.18	7.68

Mw: weight-average molecular weight.

Mn: number- average molecular weight.

Mz: Z-molecular weight.

Mw/Mn: the polydispersity of polysaccharides.

^—^Not detected in the polysaccharide.

**Figure 8:**
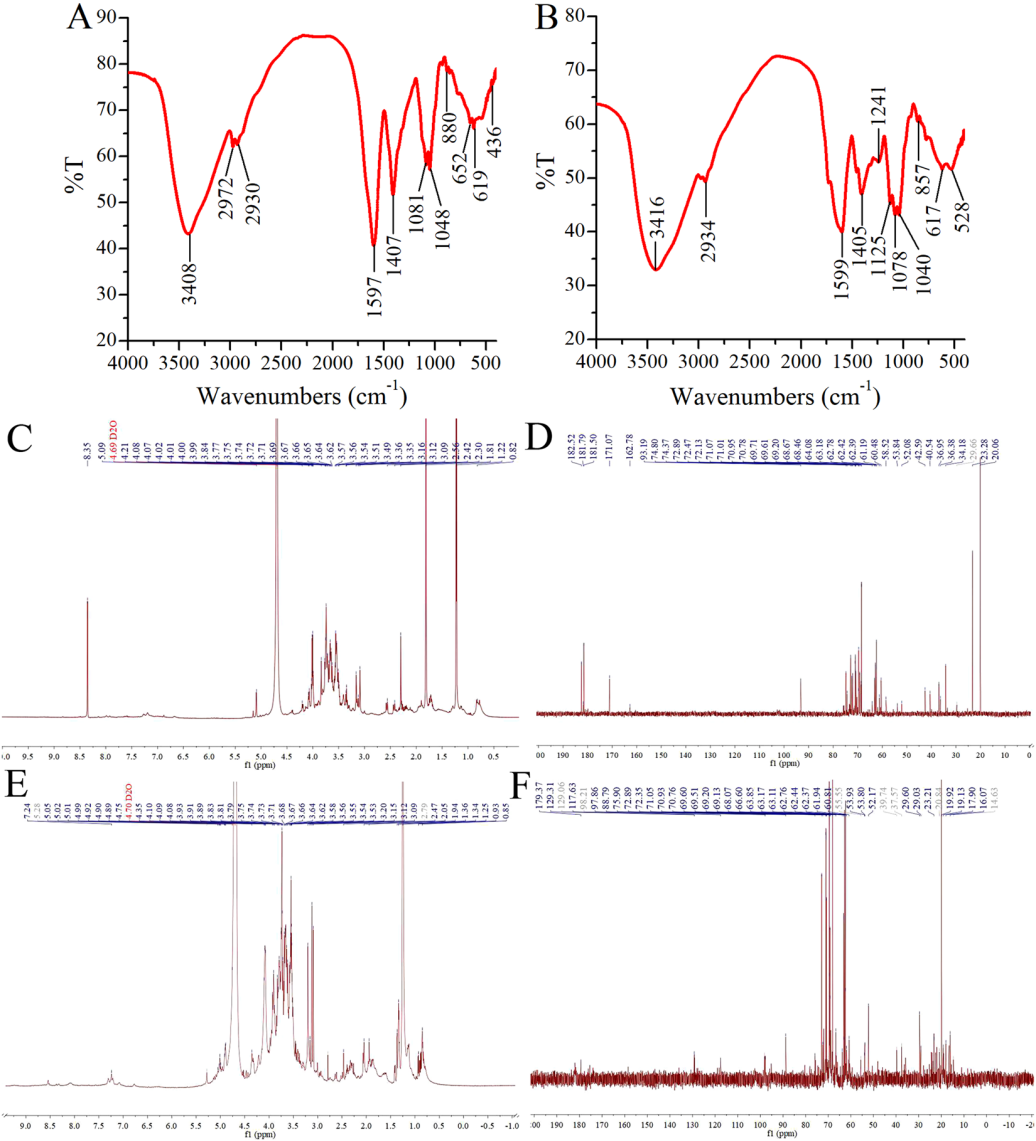
FT-IR spectra analysis of (**A**) Al-DPS and (**B**) En-DPS, and (**C**) ^1^H NMR and (**D**) ^13^C NMR of Al-DPS as well as (**E**) ^1^H NMR and (**F**) ^13^C NMR of En-DPS.

FT-IR spectroscopy is typically used for the qualitative measurement of organic functional groups and investigating the vibrations of molecules and polar bonds between the different atoms. The FT-IR spectrum of Al-DPS and En-DPS were presented in Fig. 8A,B. A strong and broad absorption peak at approximately 3400 cm^−1^ for O-H stretching vibrations was observed^17^. The peak at about 2930 cm^−1^ was ascribed to C-H stretching vibrations^18^. Moreover, the strong extensive absorption in the region of 900–1200 cm^−1^ for coupled C-O and C-C stretching and C-OH bending vibrations in the Al-DPS and En-DPS indicated the characteristic absorptions of polysaccharides^19^. There was a band at 1400 cm^−1^ due to C=O (-COOH) stretching vibration^20^. The presence of absorption band at 880 cm^−1^ in Al-DPS and 857 cm^−1^ in En-DPS manifested that Al-DPS and En-DPS were respectively typical polysaccharides linked by β- and α-type glycosidic bonds^21^.

In the ^1^H and ^13^C NMR spectrum of Al-DPS (Fig. 8C,D), dense signals distributed in the range of 3.0–5.0 ppm and 60–110 ppm indicating the representative peaks of carbohydrate^22^. The chemical shifts from 160 to 180 ppm in the ^13^C NMR spectrum of Al-DPS and En-DPS were attributed to uronic acids, which identified with the results of HPLC^23^. The signals found in the anomeric carbon region at δ 97.86 and 98.21 in the ^13^C NMR spectrum of En-DPS (Fig. 8F) confirmed the glucosyl linkage was α form. Furthermore, the anomeric signals appeared at 5.01, 5.02, 5.05 and 5.28 ppm (Fig. 8E) further indicated that the existence of α-glycosidic residues in En-DPS, which was consistent with those of the previous FT-IR analysis^23,24^. There was no signals at 80–90 ppm in the ^1^H NMR spectrum of Al-DPS, indicating that Al-DPS did not contain a 1 → 3 glucosidic bond. In the ^1^H NMR spectrum of Al-DPS and En-DPS, the signals observed at 1.22 ppm and 1.25 ppm respectively were assigned to the methyl protons of fucose^25^.

## Discussion

For a long time, mushrooms have always been treasured and appreciated owing to their unique taste and flavor. Besides, many researchers have documented that polysaccharides isolated from edible mushrooms were corroborated to possess various physiological activities that are beneficial to people’s health. Hence, the usage of mushrooms has expanded up to a wider extent not only as food but also as pharmaceuticals and nutraceuticals^26^. Polysaccharides extracted from *D*. *indusiata*, which is a precious edible mushroom well-accepted by consumers in China and other Asian countries, exhibited many bioactivities, including antioxidant, hepatoprotective, immunomodulatory, anti-tumor and hypolipidemic effects^16,27^. However, there were few reports on the antihyperlipidemic and hepatoprotective properties of alkali and enzyme extractable polysaccharides by *D*. *indusiata* until now and this was the reason why we carried on the research.

It was known that hyperlipidemia is a systemic disease, which is mainly characterized by dyslipidemia in serum^1,28^. An elevated level of TC, TG, LDL-C, AI and reduced level of HDL-C are major factors for the development of many lipid-related diseases such as atherosclerosis, cardiovascular disease and obesity^29^. Excess LDL-C, the major transporter of TC, can gather in the blood and is easily oxidized, thus induces the occurrence of oxidative damage. Conversely, HDL-C can prevent from coronary heart disease and atherosclerosis^30^. In addition, AI is also an important criterion to examine dyslipidemia. In the study, after the replenishment of high-fat emulsion for 33-days, the levels of TC, TG and LDL-C in the serum and AI of MC group were higher than NC group. Simultaneously, HDL-C level was decreased in mice of model control group as compared to normal control group as mentioned. The results proved that hyperlipidemic mice model induced by high-fat emulsion was successful. Nevertheless, polysaccharide administration almost returned the serum lipids to normal status, suggesting that Al-DPS and En-DPS had the potential to improve dyslipidaemia against hyperlipidemia by entering the blood circulation. Reports have shown that the decreases of LDL-C were possibly in connection with the enhancement of LDL-C catabolism through hepatic receptors. And the restoration of the catabolic metabolism of TG may be due to an increased stimulation of the lipolytic activity of plasma lipoprotein lipase (LPL)^31^. Similar conclusions were acquired in many previous reports^7,32^.

Reactive oxygen species (ROS), one kind of prooxidants, are highly reactive O_2_ metabolites. Documented literature indicated that oxidative stress which is defined as an imbalance between prooxidants and antioxidants, plays a critical role in the pathogenesis of more than 100 human diseases. Under normal physiological conditions, the elimination of ROS and the balance between generation and removal of ROS are performed by intracellular antioxidants to resist the oxidative damages. Whereas when the balance between generation and removal of ROS is broken, excessive accumulation of ROS will cause oxidative stress and injuries of tissues and organs^19,33^. MDA and LPO, as superfluous lipid intermediates, are capable to disturb the antioxidant defence and provide demonstrations for the pathogenic roles of oxidative stress^20^. The antioxidant enzymes such as SOD, GSH-Px and CAT, could reflect the production of free radicals and defense against the formation of ROS under the oxidative stress^34^. At the same time, T-AOC activity could reflect and represent the capacity of the non-enzymatic antioxidant defense system in the organs^11^. In the present work, increased activities of SOD, CAT, GSH-Px and T-AOC as well as decreased contents of MDA and LPO in the mice treated with polysaccharides compared with that in the MC groups showed that either Al-DPS or En-DPS as novel bioactive compounds could tend antioxidative system in liver to be normalized, thus treating hyperlipidaemia.

It was well known that hyperlipidaemia, obesity and hyperglycemia have indispensable relationships. Glucose tolerance test indicated that the hyperlipidemic mice exhibited impaired glucose tolerance. The abnormal changes of INS content which is a hormone known to stable blood glucose levels also verified the results of glucose tolerance test. LEP produced by white adipocytes could regulate energy homeostasis and appetite^35^, and ADPN is known related to glucose metabolism, fatty acid oxidation as well as insulin sensitivity^36^. In the experiment, we found that hyperlipidemia also had an impact on the concentrations of LEP and ADPN.

It was well known that some enzymatic activities in serum have long been considered as biochemical and sensitive indicators to assess hepatic injury^37^. Increased activities of ALT, AST, ALP, LDH and CK in serum after the gavage of high-fat emulsion (MC group) compared with those of normal mice indicated hepatocytes were damaged and their transport function and membrane permeability were also altered, leading to the leakage of enzymes from the cells consequently^38^. However, a noteworthy inhibition of the activities of serum enzyme was observed after the addition of polysaccharides, suggesting that Al-DPS and En-DPS had the ability to stabilize plasma membrane, thereby recovering the impaired hepatic cells.

It was reported that high-fat diet could accelerate the formation of radicals *in vivo* and destroy the intrinsic antioxidant defense, thus induces oxidative stress. Excess free radicals react with unsaturated fatty acids, leading to the generation of lipid intermediates and the alteration of cellular membranes integrity^20^. Subsequently, lipid intermediates can further disturb the antioxidant defense in liver. The damaged permeability of cell membrane results in that ALT, AST and ALP could be leached out from hepatocytes into blood circulation^11^. Polysaccharides, as a natural antioxidant supplementation, could recover the balance between generation and removal of ROS and reduce the level of oxidative stress. Therefore, the elevated levels of ALT, AST, ALP and so on in serum could be reduced. On the other hand, the intake of high-fat emulsion increases the levels of total cholesterol and causes the disordered circulation of lipid metabolism in the blood^11^. Documented literatures have shown that the lipid-lowering effects may be related to the inhibition of cholesterol biosynthesis and increased fecal bile acid excretion^31^.

Moreover, haematoxylin-eosin staining was implemented to assess the injury of liver visually^38^. In comparison with hepatic cellular architecture of mice from the NC group, severe liver damages had occurred after injection of high-fat emulsion. As expected, the hepatic lesions were markedly ameliorated by pretreatment with polysaccharides. The results were in good agreement with the results of biochemical analysis in serum. It was well-known that the bioactive properties of polysaccharides were closely related to their structural characterizations. The En-DPS exhibited the stronger biological effect than Al-DPS may be associated with its molecular weight and configurations^20^.

## Conclusions

According to the current work, Al-DPS and En-DPS exhibited protective action against hyperlipidemia induced by high-fat emulsion as evidenced by reducing lipid profile and mitigating oxidative stress in liver, which was confirmed by histopathologic observation. The results demonstrated that Al-DPS and En-DPS played momentous roles in the prevention and treatment of hyperlipidemia related liver impairments.

## Materials and Methods

### Materials and reagents

The dried fruiting bodies of *D*. *indusiata* used in this experiment were provided by Taian Academy of Agricultural Sciences (Taian, China). The diagnostic kits for analyzing the activities of glutathione peroxidase (GSH-Px), superoxide dismutase (SOD), catalase (CAT) and total antioxidant capacity (T-AOC), the levels of total cholesterol (TC), triglycerides (TG) and nonestesterified fatty acid (NEFA) as well as the contents of lipid peroxide (LPO), malondialdehyde (MDA) were purchased from Nanjing Jiancheng Bioengineering Institute (Nanjing, China). Besides, the insulin (INS), leptin (LEP) and adiponectin (ADPN) contents were measured using commercial enzyme-linked immunosorbent assay (ELISA) kits from Jiangsu Meibiao Biological Technology Company Limited (Jiangsu, China). All the other reagents used in the work were of analytical grade and supplied by local chemical suppliers.

### Preparation of Al-DPS and En-DPS

Two kinds of polysaccharide samples were prepared on the basis of methods reported previously with slight modifications^39^. To obtain Al-DPS or En-DPS, the powders of *D*. *indusiata* fruiting bodies pulverized by a disintegrator were extracted with NaOH solution (0.5 mol/L, 1:10, w/v) at 85 °C for 5 h or snailase solution (4%, 1:4, w/v) at 38 °C for 4 h, respectively. The extraction steps above were repeated three times and supernatants centrifuged at 3000 r/min for 10 min were mixed with four-fold volumes of 95% ethanol (v/v) and kept overnight at 4 °C. The precipitate separated by centrifugation was deproteinated by employing the Sevag method^40^. Finally, after being dialyzed and freeze-dried (Labconco, USA), Al-DPS and En-DPS were prepared successfully and used for further experiments. The percentage of polysaccharides yield was calculated according to the following formula^41^.

1 $${\rm{Polysaccharide}}\,{\rm{yield}}\,( \% )={{\rm{W}}}_{1}/{{\rm{W}}}_{0}\times 100$$

where W_1_ was the weights of Al-DPS or En-DPS (g) and W_0_ was powders sample weights (g).

### Acute toxicity study

According to a modified method previously described by reports, acute toxicity experiment was performed^42,43^. Forty-two male Kunming strain mice were divided into seven groups at random including one control group and six dosage groups. After fasted overnight, the mice in dosage groups were gavaged with Al-DPS or En-DPS at the dose of 800, 1000 and 1200 mg/kg respectively and control group received isometric normal saline solution. Normal feed and free access to drinking water were given to all the mice. After dosing, the mice were kept continuously observations for gross behavioral changes, toxic symptoms and mortality for 14 days (with special attention given during the first 24 h). At the end of the experiment, all the mice were weighted and sacrificed by euthanasia. The alamine aminotransferase (ALT) and aspertate aminotransferase (AST) of serum were measured. The liver was quickly excised, weighed and macroscopically examined. Moreover, haematoxylin-eosin (H&E) staining of liver was performed.

### Animal experiments

The high-fat emulsion was prepared according to a reported method^34^. Lard oil (25 g) heated to 100 °C was mixed thoroughly with 10 g cholesterol, 1 g methylthiouracil and 25 mL of Tween-80, which was oil phase. Simultaneously, the water phase contained 30 mL distilled water, 20 mL propylene glycol and 2 g sodium deoxycholate. In the end, the high-fat emulsion was finished after the mix of oil phase and water phase before animal administration.

Seventy Kunming strain mice (20 ± 2 g, male) were purchased from Taibang Biological Products Ltd. Co. (Taian, China) and maintained at an animal room with controlled temperature (25 ± 2 °C), humidity (55 ± 5%) and a 12 h light/dark cycle with free access to water and standard food. All experiments were performed in accordance with the Regulations of Experimental Animal Administration issued by the State Committee of Science and Technology of the People’s Republic of China.

After one-week acclimatization period, all mice were randomly distributed into seven groups with ten mice in each group containing two Al-DPS groups (400, 200 mg/kg/d), two En-DPS groups (400, 200 mg/kg/d), as well as three control groups including normal control group (NC), model control group (MC), and positive control group (PC). To induce hyperlipidemia, all the mice were administered with gastric injection of high-fat emulsion every day except NC group which received isometric saline solution. Subsequently, the mice were gavaged with different dosage polysaccharides, NC and MC groups with isometric saline solution and PC group with simvastatin (200 mg/kg/d).

After a 33-consecutive gavage, all mice were weighed and sacrificed under anaesthetic treatment after overnight fasting following the last administration. Then serum sample was separated from the blood through centrifugation (6000 r/min, 10 min). The levels of TC, TG, low density lipoprotein cholesterol (LDL-C) and high-density lipoprotein cholesterol (HDL-C) as well as the activities of ALT, AST, alkaline phosphatase (ALP), lactate dehydrogenase (LDH), creatine kinase (CK) were analyzed using an automated biochemical analyzer (Shenzhen, China).

The tissue of liver was immediately excised, washed with ice-cold saline, weighed and homogenized (1:9, g/mL) in phosphate buffer solutions (PBS, 0.2 mol/L, pH 7.4). The homogenates were centrifuged (4000 r/min) at 4 °C for 10 min and the supernatants were collected for further biochemical analysis. Hepatosomatic index (HI) and atherogenic index (AI) were calculated according to the following formulas^44^.

2 $${\rm{HI}}\,( \% )={\rm{liver}}\,{\rm{weight}}/{\rm{body}}\,{\rm{weight}}\times 100$$

3 $${\rm{AI}}=(\mathrm{TC} \mbox{-} \mathrm{HDL} \mbox{-} {\rm{C}})/\mathrm{HDL} \mbox{-} {\rm{C}}$$

In addition, the fresh liver was washed with normal saline instantaneously, fixed in 4% formalin and embedded in paraffin. Subsequently, the tissue was cut into 5 μm slices for H&E staining^45^. In the end, all the sections were observed under a microscope (400 × magnification) for histological analyses.

### Glucose tolerance test

Before sacrifice, we tested blood glucose level from mice tail vein after fasting for 12 h by a glucometer and set it as the blood sugar at time 0 min. Subsequently, the mice were given glucose solution of 1.5 g/kg through intraperitoneal injection and blood glucose was detected at 30, 60, 90, and 120 min, respectively. Ultimately, we drew the curve of blood glucose with time according to the records.

### Structural characterization of Al-DPS and En-DPS

#### Molecular weight determination

Briefly, the molecular weight of Al-DPS and En-DPS were measured by high performance gel permeation chromatography (HPGPC) that was operated with a HPLC system (Agilent 1260, Agilent Technologies, CA, USA) equipped with a Shodex SB-806HQ column (8 mm × 300 mm, Showa Denko K.K., Tokyo, Japan) and a differential refractive index detector. A series of standard dextrans (Sigma) with different molecular weights were used to establish the calibration curve^20^.

#### Monosaccharide composition analysis

The monosaccharide compositions were determined by high performance liquid chromatography (HPLC, Ultimate 3000) equipped with Xtimate C18 column (4.6 × 200 mm, 5 um) at 30 °C with a flow rate of 1.0 mL/min. By the comparisons with standard sugars, the monosaccharide compositions of samples were assayed.

#### Fourier transform infrared spectroscopy (FT-IR) analysis

Two kinds of polysaccharides were mixed with potassium bromide (KBr) powder severally and pressed into pellets for the FT-IR spectral measurement with a wavenumber range of 4000–400 cm^−1^, which was performed through an infrared spectrometer (Nicolet 6700, Thermo Fisher Scientific, USA)^46^.

#### Nuclear magnetic resonance (NMR) spectroscopy analysis

After dissolved in D_2_O, the ^1^H and ^13^C NMR spectra of Al-DPS and En-DPS were recorded via a Bruker DRX-500 spectrometer at 25 °C.

#### Statistical analysis

The data were expressed as the mean ± standard deviations (S.D.) and significant differences between the experimental groups were determined by the one-way ANOVA followed by Duncan-test (SPSS 19.0 software package, USA). Meanwhile, P < 0.05 was considered to be statistically significant.

### Compliance with ethical standards

The experiments were performed as approved by the Institutional Animal Care and Use Committee of Shandong Agricultural University, and in accordance with the Animals (Scientific Procedures) Act 1986 (amended 2013).
